# Prediction of disease progression in patients with COVID-19 by artificial intelligence assisted lesion quantification

**DOI:** 10.1038/s41598-020-79097-1

**Published:** 2020-12-16

**Authors:** Yuehua Li, Kai Shang, Wei Bian, Li He, Ying Fan, Tao Ren, Jiayin Zhang

**Affiliations:** 1 Institute of Diagnostic and Interventional Radiology, Shanghai Jiao Tong University Affiliated Sixth People’s Hospital, #600, Yishan Rd, Shanghai, 200233 China; 2 Department of Respiratory Medicine, Shanghai Jiao Tong University Affiliated Sixth People’s Hospital, #600, Yishan Rd, Shanghai, China; 3 Department of Nephrology, Shanghai Jiao Tong University Affiliated Sixth People’s Hospital, #600, Yishan Rd, Shanghai, China

**Keywords:** Computational biology and bioinformatics, Medical research

## Abstract

To investigate the value of artificial intelligence (AI) assisted quantification on initial chest CT for prediction of disease progression and clinical outcome in patients with coronavirus disease 2019 (COVID-19). Patients with confirmed COVID-19 infection and initially of non-severe type were retrospectively included. The initial CT scan on admission was used for imaging analysis. The presence of ground glass opacity (GGO), consolidation and other findings were visually evaluated. CT severity score was calculated according to the extent of lesion involvement. In addition, AI based quantification of GGO and consolidation volume were also performed. 123 patients (mean age: 64.43 ± 14.02; 62 males) were included. GGO + consolidation was more frequently revealed in progress-to-severe group whereas pure GGO was more likely to be found in non-severe group. Compared to non-severe group, patients in progress-to-severe group had larger GGO volume (167.33 ± 167.88 cm^3^ versus 101.12 ± 127 cm^3^, p = 0.013) as well as consolidation volume (40.85 ± 60.4 cm^3^ versus 6.63 ± 14.91 cm^3^, p < 0.001). Among imaging parameters, consolidation volume had the largest area under curve (AUC) in discriminating non-severe from progress-to-severe group (AUC = 0.796, p < 0.001) and patients with or without critical events (AUC = 0.754, p < 0.001). According to multivariate regression, consolidation volume and age were two strongest predictors for disease progression (hazard ratio: 1.053 and 1.071, p: 0.006 and 0.008) whereas age and diabetes were predictors for unfavorable outcome. Consolidation volume quantified on initial chest CT was the strongest predictor for disease severity progression and larger consolidation volume was associated with unfavorable clinical outcome.

## Introduction

Coronavirus disease 2019 (COVID-19) is a worldwide pandemic which firstly outbroke in Wuhan, China in December 2019 and spread to hundreds of nations since then^1^. Although a large proportion of patients with COVID-19 disease were mild cases, the rate of severe and critical cases was not low and the mortality could be as high as 21%^2,3^.

Chest computed tomography (CT) has been used as the first-line imaging modality for prompt diagnosis and monitoring disease course of COVID-19 pneumonia^4^. Compared to reverse transcription polymerase chain reaction (RT-PCR), chest CT has very high sensitivity to identify COVID-19 pneumonia^5^. The extent of involved pulmonary lesions was also found to be associated with unfavorable clinical outcome^6^.

In addition, artificial intelligence (AI) has emerged as a useful tool in terms of quantitative evaluation of pulmonary diseases, such as emphysema^7^. Compared to visual analysis, it allows more precise quantification of pulmonary lesions based on different attenuation thresholds. Moreover, according to recent studies, AI was found to be helpful for distinguishing COVID-19 from pneumonia of other origin through Chest CT with high diagnostic accuracy, and predict respiratory complications of COVID-19^8–10^. However, whether AI-derived lesion quantification on chest CT was related to disease progression remained unclear. Therefore, the current study aims to investigate the value of AI assisted quantification for prediction of disease severity progression and clinical outcome, and compare it with conventional CT parameters evaluated by radiologist.

## Material and methods

### Patient population

The institutional review board of Shanghai Jiao Tong University Affiliated Sixth People’s Hospital approved this retrospective study with a waiver of informed consent. All methods in the current study was carried out in accordance with relevant guidelines and regulations. From February 10th, 2020 to April 9th, 2020, hospitalized patients with confirmed COVID-19 infection (diagnosed by RT-PCR assay with throat swab) were retrospectively included. The RT-PCR tests were performed by using TaqMan One-Step RT-PCR Kits from Shanghai Huirui Biotechnology Co., Ltd. The diagnosis was made if any of the RT-PCR result was positive when multiple tests were performed. The exclusion criteria were: (1) the image quality of chest CT was significantly impaired so that AI based quantification was not feasible; (2) patients had superimposed infection of other pathogens; (3) patients were initially classified as severe or critical type on admission. The initial CT scan on admission was used for further analysis.

### Chest CT protocol

All patients were scanned with a 128-slice multi-detector CT (Revolution Maxima, GE Medical Systems, Milwaukee, US) in supine position with inspiratory breath hold. The main acquisition parameters were listed as follow: tube voltage = 120 kVp, tube current was automatically modulated (150 mA–350 mA), slice thickness = 1.25 mm, slice interval = 1 mm, matrix = 512 * 512, field of view = 350 mm * 350 mm. Two datasets with different kernels were reconstructed for the image interpretation of lung (sharp kernel, Lung, GE Medical Systems) and mediastinum (smooth kernel, Stnd, GE Medical Systems).

### Visual analysis of chest CT

All datasets were reconstructed with lung kernel and soft kernel for evaluation of pulmonary parenchyma and mediastinum. Lung window was set as a level of − 600HU and width of 1200HU whereas mediastinal window was set a level of 40HU and width of 350HU. The presence of following lesions were visually assessed: (1) ground glass opacity (GGO), which was defined as hazy increased attenuation with preserved margins of bronchus and vasculature^11^; (2) consolidation, which was defined as opacification with obscured margins of bronchus and vasculature; (3) reticulation; (4) nodules; (5) lymphadenopathy; (6) pleural effusion; (7) other abnormalities.

For semi-quantitative analysis, one previously reported CT score system was employed^12^. In brief, each lung was divided into three zones: upper (above the carina), middle (between carina and inferior pulmonary vein), and lower (below the inferior pulmonary vein) zones. Each lung zone was assigned a score that was based on the extent of involvement: score 0, 0% involvement; score 1, < 25% involvement; score 2, 25–50% involvement; score 3, 50–75% involvement; and score 4, ≥ 75% involvement. The summed score on per-patient level indicated overall lung involvement (maximal CT score for both lungs was 24)^12^.

Two chest radiologists (with 6-year and 14-year experience of chest imaging) independently evaluated all patients without the knowing of clinical characteristics and prognosis. Any disagreement between two observers was resolved by consensus.

### AI assisted lesion quantification

Lesion quantification was performed using a commercially available software (LungDoc, version 1.19.1, ShuKun Network Technology, Beijing, China). In the recognition of pneumonia regions, the semantic segmentation technology based on deep learning was used to perform one-time segmentation extraction of the pneumonia regions in the input lung parenchyma. The network used a deep convolutional neural network based on residual structure UNet, of which details were given in online supplemental materials. The volumes of GGO and consolidation were automatically measured according to different attenuation thresholds (− 750HU ~ − 300HU for GGO, − 300HU ~ 50HU for consolidation)^13^. The percentages of GGO volume as well as consolidation volume versus whole lung volume were also recorded.

Two chest radiologists (with 6-year and 14-year experience of chest imaging) independently supervised the lesion quantification and manual adjustment of lesion regions was made when necessary. Any disagreement between two observers was resolved by consensus.

### Disease severity and clinical outcome

Disease severity was evaluated according to the 6th edition of diagnosis and treatment protocols of pneumonia caused by novel coronavirus, issued by Chinese centers for disease control and prevention^14^. Patients with confirmed diagnosis of COVID-19 were classified into four types as described below: (1) mild, patients with mild symptoms and no imaging finding of pneumonia; (2) moderate, patients with fever, respiratory symptoms and imaging findings of pneumonia; (3) severe, patients met any of the following conditions (a. respiratory distress, respiratory rate ≥ 30 times/min; b. SpO_2_ < 93% at rest; c. PaO2/FiO2 ≤ 300 mmHg; d. rapid progression of disease involvement [more than 50%] on chest CT within 24 ~ 48 h ); (4) critical, patients met any of the following conditions (a. respiratory failure and need mechanical assistance; b. shock; c. “extra pulmonary” organ failure, d. intensive care unit [ICU] is needed). For further analysis, patients were grouped as “non-severe” (classified as mild or moderate type) and “progress-to-severe” (classified as severe or critical type) according to the most severe classification during hospitalization.

In addition, the mortality rate and rate of critical conditions (a. respiratory failure and need mechanical assistance; b. shock; c. “extra pulmonary” organ failure, d. ICU stay) were also recorded. Patients were considered event-free if they survived without occurrence of the above critical conditions.

### Statistical analysis

Statistical analysis was performed with a commercially available statistical software (MedCalc Statistical Software version 15.2.2, MedCalc Software BVBA, Ostend, Belgium). Quantitative variables were expressed as mean ± standard deviation (SD). Student’s t test was used for normally distributed data, and the Mann–Whitney U test for non-normally distributed data. Categorical variables were reported as count (%), and compared by the Fisher exact test or chi-square test. Interobserver agreement of CT semi-quantitative score and AI assisted quantification was assessed by intraclass correlation coefficient (ICC). The Dice coefficient was used to evaluate the performance of the deep learning algorithms. Logistic regression model was used to calculate the area under the receiver operating characteristic (ROC) curve for different CT parameters. ROC analysis was performed by the method developed by Hanley and McNeil^15^. The area under curve (AUC) of each parameter was calculated and the optimal cutoff values for all parameters were determined by the Youden index, the maximum sum of sensitivity and specificity. To ascertain the associations of CT-derived semi-quantitative and quantitative parameters with disease severity progression and clinical outcome, we performed univariate and multivariate logistic regression analyses, which was performed by using the “stepwise” approach. The model included variables with p < 0.20 in the univariate analysis. A two-tailed P < 0.05 was considered statistically significant.

## Results

### Patient characteristics

**Figure 1:**
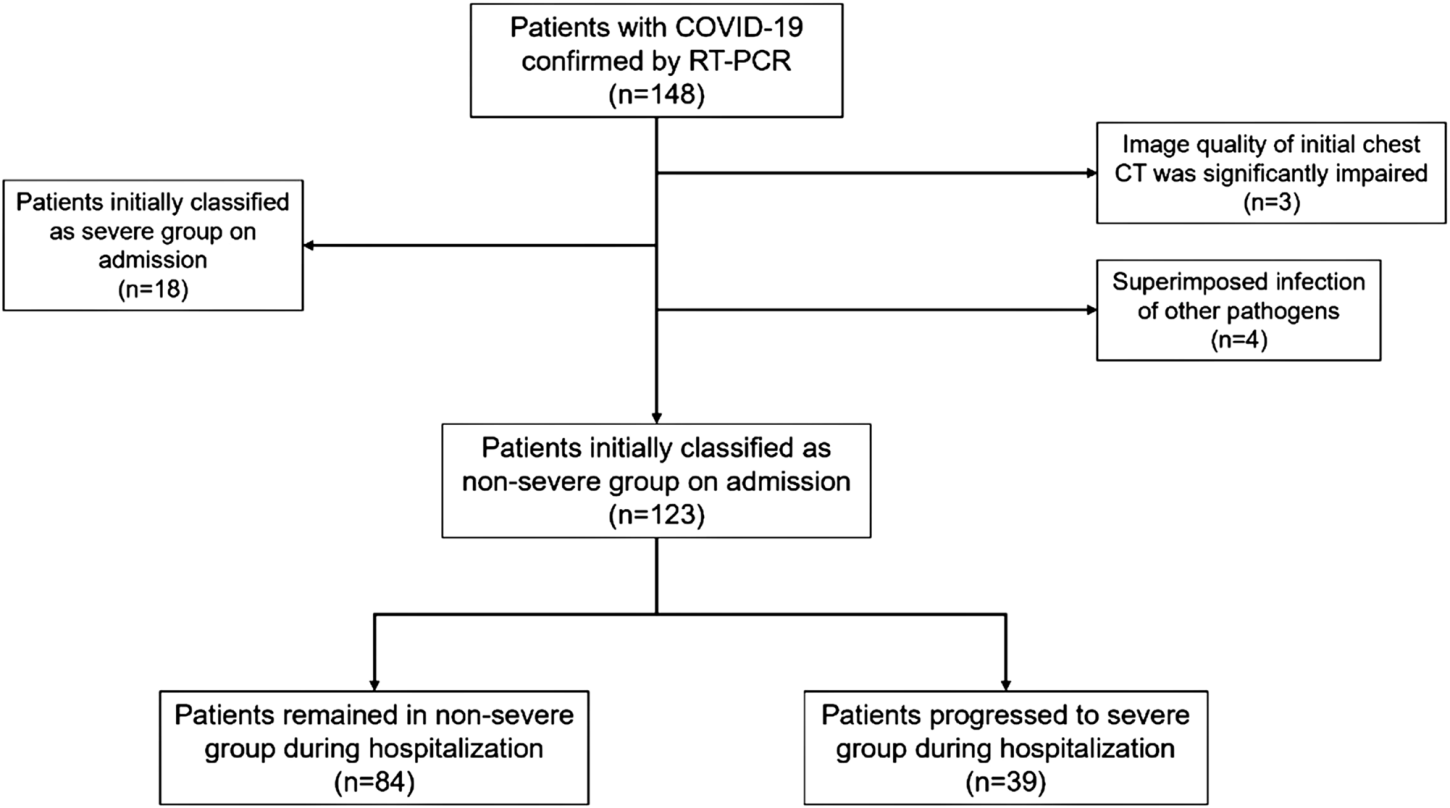
Flow chart of inclusion and exclusion. *COVID-19* Coronavirus disease 2019, *CT* computed tomography, *RT-PCR* reverse transcription polymerase chain reaction.

From February 10th, 2020 to April 9th, 2020, 148 patients with confirmed COVID-19 infection were retrospectively reviewed. Three patients were excluded due to significantly impaired image quality of chest CT whereas 4 patients were excluded because of having superimposed infection of other pathogens. Moreover, another 18 patients who were classified as severe or critical type were also ruled out (Fig. 1). Finally, 123 patients [mean age: 64.43 ± 14.02 (range 30–93) years, 62 males] were included in the further analysis. All patients were followed up at a mean time of 23.2 ± 11.7 days. For patients from progressed to severe group, the mean interval between admission and disease progression was 11.3 ± 3.4 days. Detailed clinical characteristics were given in Table 1.

**Table 1:** Demographic and clinical characteristics of the patients.

	All patients (n = 123)	Non-severe group (n = 84)	Progress-to-severe group (n = 39)	p value*
Age	64.43 ± 14.02	61.01 ± 13.438	71.79 ± 12.444	< 0.001
Men	62/123 (50.41%)	36/84 (42.86%)	26/39 (66.67%)	0.014
Clinical symptoms				
No symptom	13/123 (10.57%)	10/84 (11.90%)	3/39 (7.69%)	0.753
Fever	80/123 (65.04%)	53/84 (63.10%)	27/39 (69.23%)	0.507
Cough	76/123 (61.79%)	53/84 (63.10%)	23/39 (58.97%)	0.662
Sputum production	30/123 (24.39%)	22/84 (26.19%)	8/39 (20.51%)	0.495
Shortness of breath	59/123 (47.97%)	34/84 (40.48%)	25/39 (64.10%)	0.015
Preexisting conditions				
Diabetes	29/123 (23.58%)	14/84 (16.67%)	15/39 (38.46%)	0.008
Hypertension	59/123 (47.97%)	37/84 (44.05%)	22/39 (56.41%)	0.202
COPD	12/123 (9.76%)	4/84 (4.76%)	8/39 (20.51%)	0.006
Cardiovascular diseases	26/123 (21.14%)	14/84 (16.67%)	12/39 (30.77%)	0.063
Intensive care unit	33/123 (26.83%)	0/84 (0.00%)	33/39 (84.62%)	< 0.001
Mechanical ventilation	7/123 (5.69%)	0/84 (0.00%)	7/39 (17.95%)	< 0.001
Septic shock	11/123 (8.94%)	0/84 (0.00%)	11/39 (28.21%)	< 0.001
ARDS	6/123 (4.88%)	0/84 (0.00%)	6/39 (15.38%)	< 0.001
Duration of hospitalization, days	24.30 ± 11.50	23.45 ± 10.19	26.16 ± 13.94	0.288
Clinical outcome				
Discharge from hospital	115/123 (93.50%)	84/84 (100.00%)	31/39 (79.49%)	< 0.001
Death	5/123 (4.07%)	0/84 (0.00%)	5/39 (12.82%)	0.003
Hospitalization	3/123 (2.44%)	0/84 (0.00%)	3/39 (7.69%)	0.030

*ARDS* acute respiratory distress syndrome, *COPD* Chronic obstructive lung disease.

### Initial chest CT findings between non-severe and progress-to-severe group

During hospitalization, 39 patients progressed to severe group while 84 patients remained in non-severe group. Overall, pure GGO and GGO + consolidation were the two predominant findings of initial chest CT (Table 2). Other findings, such as reticulation, nodule, cavitation, were infrequently presented. As for subgroup analysis, GGO + consolidation was more frequently revealed in progress-to-severe group whereas pure GGO was more likely to be found in non-severe group (Table 2). Moreover, patients in progress-to-severe group had higher incidence of pleural effusion, nodule and larger number of involved lobes (Table 2).

**Table 2:** Initial chest CT findings between non-severe and progress-to-severe group.

	All patients (n = 123)	Non-severe group (n = 84)	Progress-to-severe group (n = 39)	p value*
Pure GGO (%)	49/123 (39.84%)	42/84 (50.00%)	7/39 (17.95%)	0.001
GGO + Consolidation (%)	63/123 (51.22%)	33/84 (39.29%)	30/39 (76.92%)	< 0.001
Pure consolidation (%)	2/123 (1.63%)	1/84 (1.19%)	1/39 (2.56%)	0.535
Reticulation (%)	15/123 (12.19%)	7/84 (8.33%)	8/39 (20.51%)	0.055
Nodule (%)	6/123 (4.88%)	0/84 (0.00%)	6/39 (15.38%)	0.001
Cavitation (%)	4/123 (3.25%)	1/84 (1.19%)	3/39 (7.69%)	0.094
Emphysema (%)	14/123 (11.38%)	7/84 (8.33%)	7/39 (17.95%)	0.118
Pleural effusion (%)	33/123 (26.83%)	12/84 (14.29%)	21/39 (53.85%)	< 0.001
Lymphadenopathy (%)	8/123 (6.50%)	5/84 (5.95%)	3/39 (7.69%)	0.708
Lobes involved	3.70 ± 1.69	3.46 ± 1.79	4.21 ± 1.32	0.030
CT severity score	6.75 ± 4.79	5.82 ± 4.19	8.74 ± 5.41	0.004
AI assisted quantification				
GGO volume (cm^3^)	122.11 ± 143.90	101.12 ± 127.00	167.33 ± 167.88	0.013
Consolidation volume (cm^3^)	17.48 ± 39.28	6.63 ± 14.91	40.85 ± 60.40	< 0.001
GGO volume percentage (%)	4.53 ± 5.90	3.33 ± 4.72	7.11 ± 7.28	0.001
Consolidation volume percentage (%)	1.07 ± 2.36	0.56 ± 2.05	2.17 ± 2.62	< 0.001

*AI* artificial intelligence, *GGO* ground glass opacity.

**Figure 2:**
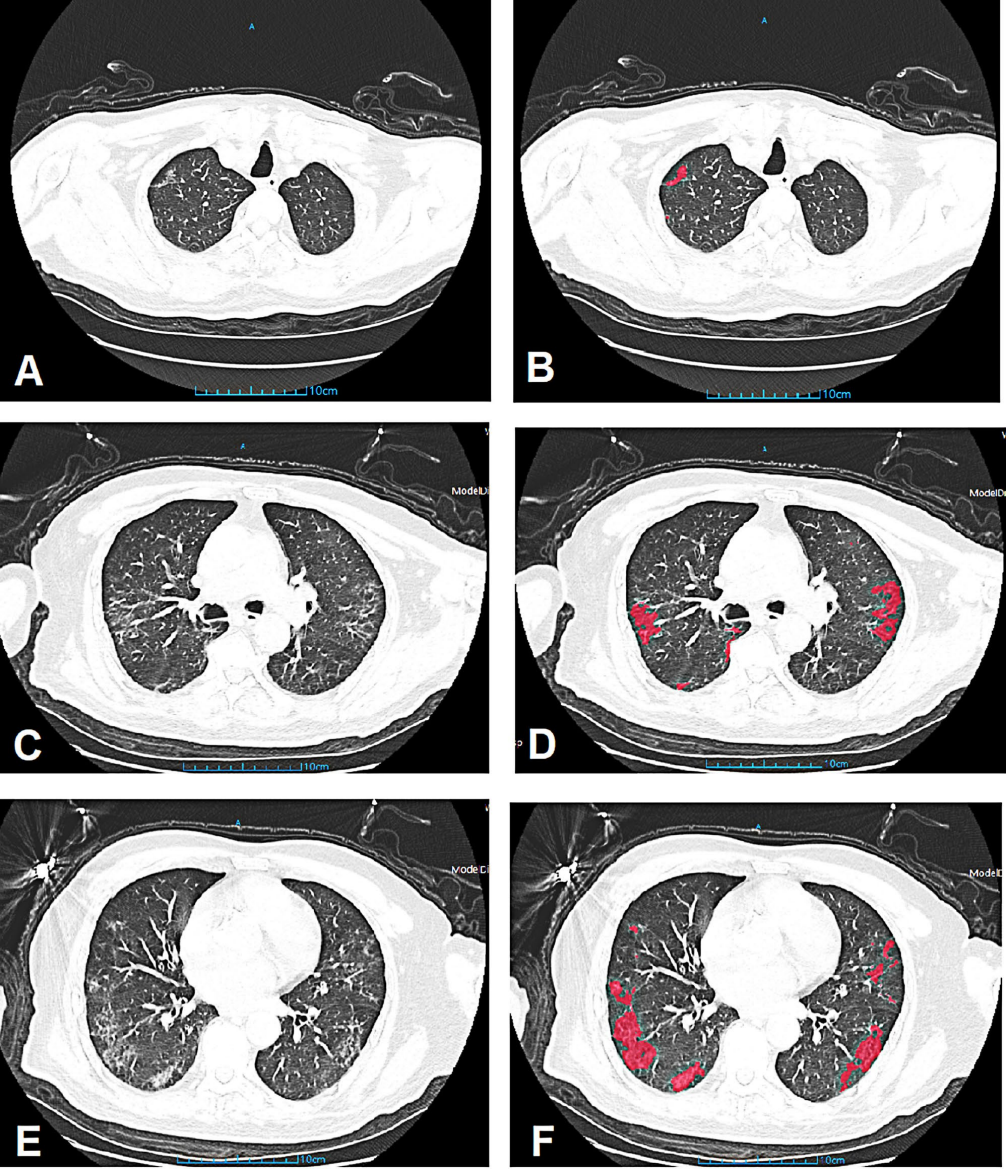
Representative case of a 78-year-old male patient with COVID-19 from non-severe group. Initial chest CT on admission showed multiple GGO lesions from upper (**A**,**B**), middle (**C**,**D**) and lower (**E**,**F**) zones. The CT severity score was 6. The GGO volume and GGO volume percentage were measured as 103.93 cm^3^ and 3.09%. *COVID-19* Coronavirus disease 2019, *CT* computed tomography, *GGO* ground glass opacity.

**Figure 3:**
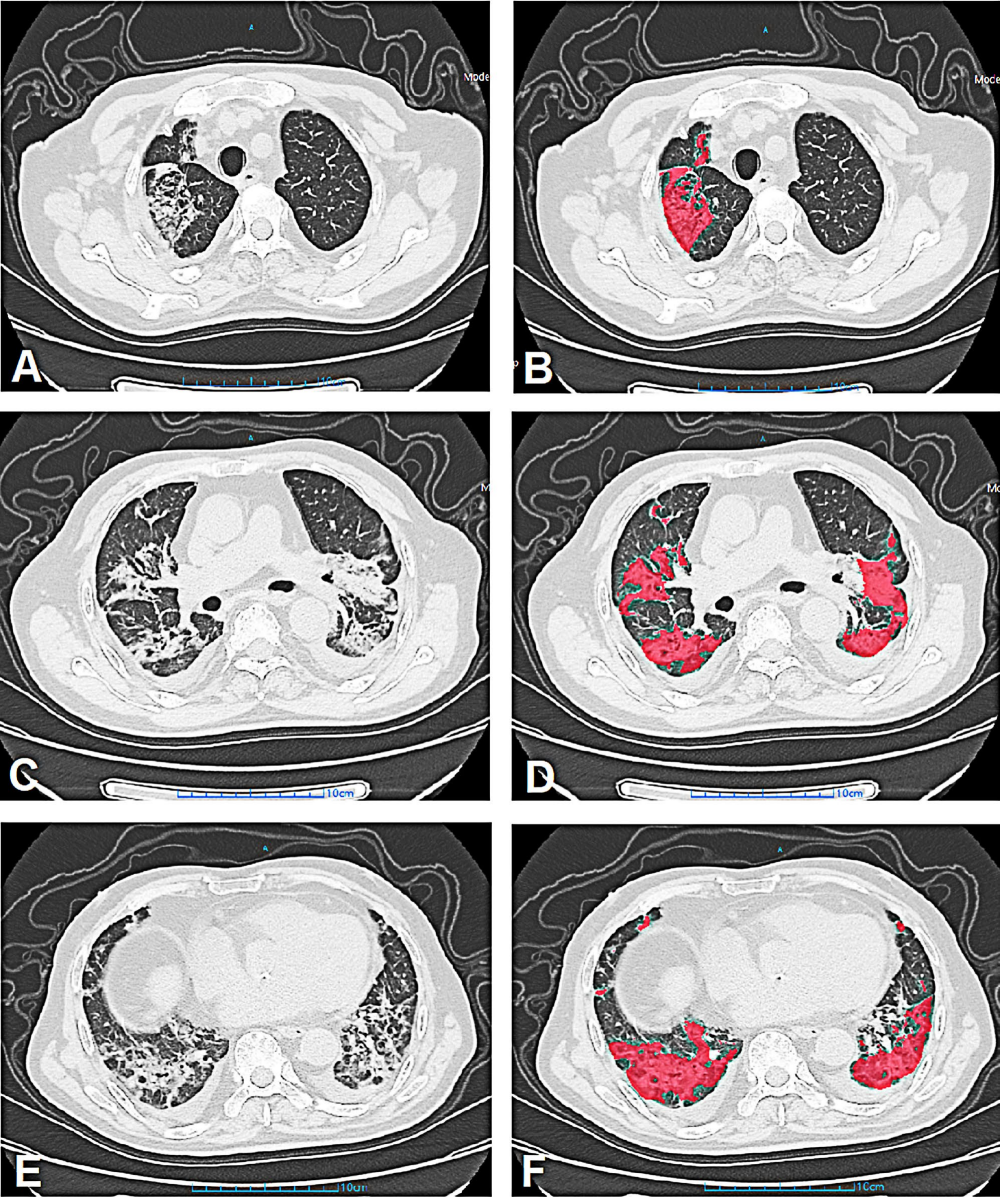
Representative case of a 78-year-old male patient with COVID-19 from progress-to-severe group. Initial chest CT on admission showed multiple GGO lesions from upper (**A**,**B**), middle (**C**,**D**) and lower (**E**,**F**) zones. The CT severity score was 11. The GGO volume, GGO volume percentage, consolidation volume and consolidation volume percentage were measured as 252.56 cm^3^, 15.08%, 90.45 cm^3^ and 5.4% respectively. *COVID-19* Coronavirus disease 2019, *CT* computed tomography, *GGO* ground glass opacity.

In terms of CT semi-quantitative score and AI assisted quantification, both methods had excellent inter-observer agreement (ICC for CT score: 0.897, 95% CI 0.875–0.921; ICC for GGO quantification: 0.931, 95% CI 0.899–0.958; ICC for consolidation quantification: 0.972, 95% CI 0.951–0.998; all p < 0.001). The regions of lesion (pneumonia) were reliably segmented by AI assisted quantification, with a Dice coefficient of 0.845 (CI 0.751–0.944, p = 0.005). Compared to non-severe group, patients in progress-to-severe group had larger GGO volume as well as consolidation volume. The GGO volume percentage and consolidation volume percentage was also significantly higher in severe group (Table 2, Figs. 2 and 3).

### Correlation of CT-derived parameters with disease severity progression and clinical outcome

**Figure 4:**
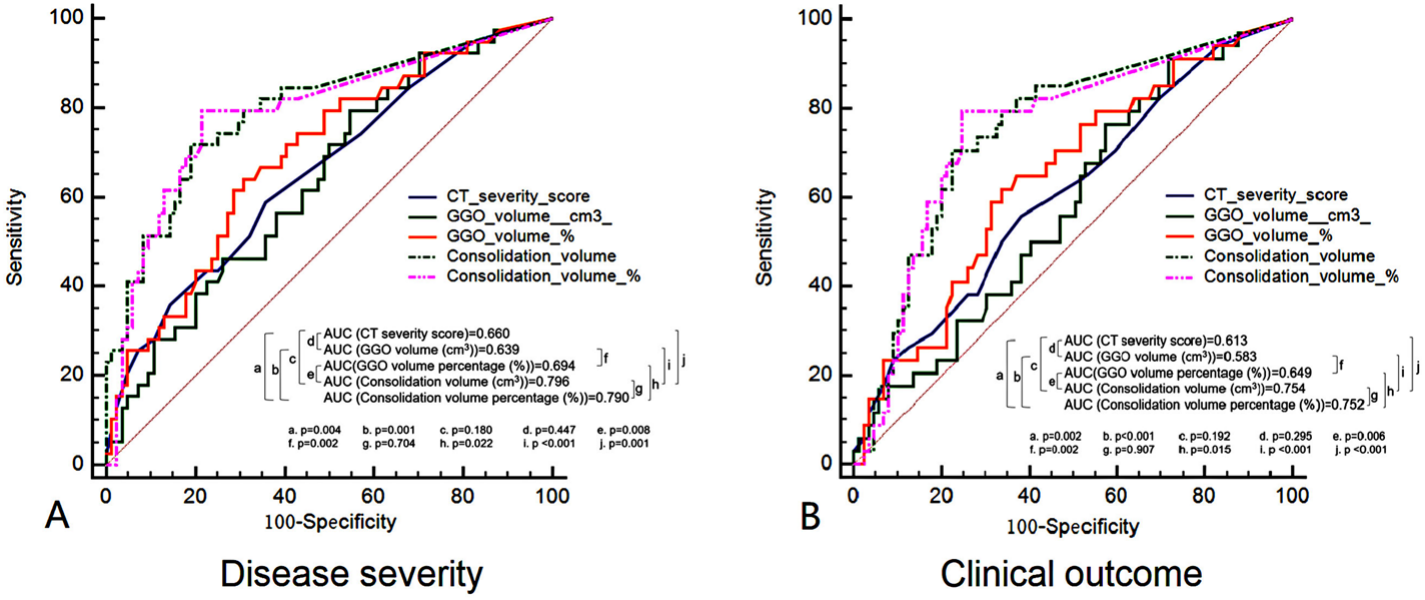
ROC curve analysis of CT-derived parameters for predicting disease severity progression (**A**) and clinical outcome (**B**). Disease severity progression was defined as the prediction of progress-to-severe group. Clinical outcome was defined as the prediction of occurrence of critical events (a. respiratory failure and need mechanical assistance; b. shock; c. “extra pulmonary” organ failure, d. ICU stay). *AUC* area under curve, *CT* computed tomography, *GGO* ground glass opacity, *ICU* intensive care unit, *ROC* receiver operating characteristic.

ROC curve analysis and logistic regression was performed to determine the predictive value of CT-derived parameters for prediction of disease severity progression. As shown by ROC analysis, consolidation volume and consolidation volume percentage had the largest AUC in discriminating non-severe from progress-to-severe group (Fig. 4). AI assisted quantification outperformed CT semi-quantitative score for identifying patients progressing to severe group with higher diagnostic accuracy, sensitivity and specificity (Table 3). According to univariate analysis, consolidation volume and consolidation volume percentage were the strongest predictors for disease severity progression among all imaging parameters, followed by CT severity score and GGO volume percentage. After the adjustment by multivariate regression, consolidation volume and age remained as the strongest predictors (hazard ratio: 1.053 and 1.071, p: 0.006 and 0.008) (Table 4).

**Table 3:** ROC curve analysis of CT-derived parameters for predicting disease severity progression and clinical outcome.

	Best cutoff	AUC	95% CI	p value	Accuracy (%)	Sensitivity (%)	Specificity (%)
Disease severity progression*							
CT severity score	> 6	0.660	0.569–0.743	0.003	62.60	58.97	64.29
GGO volume (cm^3^)	> 37.29	0.639	0.548–0.724	0.008	43.90	79.49	45.24
GGO volume percentage (%)	> 2.85	0.694	0.605–0.774	< 0.001	62.60	64.10	69.05
Consolidation volume (cm^3^)	> 5.84	0.796	0.714–0.863	< 0.001	78.05	71.79	80.95
Consolidation volume percentage (%)	> 0.14	0.790	0.707–0.858	< 0.001	78.86	79.49	78.57
**Clinical outcome#**							
CT severity score	> 6	0.613	0.521–0.700	0.047	60.16	55.88	61.80
GGO volume (cm^3^)	> 15.29	0.583	0.490–0.671	0.139	45.53	91.18	28.09
GGO volume percentage (%)	> 2.85	0.649	0.558–0.733	0.007	60.16	61.76	66.29
Consolidation volume (cm^3^)	> 5.84	0.754	0.668–0.827	< 0.001	74.80	70.59	77.53
Consolidation volume percentage (%)	> 0.14	0.752	0.666–0.826	< 0.001	76.42	79.41	75.28

*AUC* area under curve, *CT* computed tomography, *CI* confidence interval, *GGO* ground glass opacity, *ICU* intensive care unit, *ROC* receiver operating characteristic.

*Disease severity progression was defined as the prediction of progress-to-severe group.

^#^Clinical outcome was defined as the prediction of occurrence of critical events (a. respiratory failure and need mechanical assistance; b. shock; c. “extra pulmonary” organ failure, d. ICU stay).

**Table 4:** Univariate and multivariate analysis of CT-derived parameters for prediction of disease severity progression and clinical outcome.

Variables	Univariate analysis			Multivariate analysis		
	Odds ratio	95% CI	P value	Odds ratio	95% CI	P value
Disease severity progression*						
Age	1.071	1.033–1.109	< 0.001	1.071	1.018–1.126	0.008
Diabetes	0.303	0.128–0.717	0.007	0.392	0.128–1.198	0.100
Hypertension	1.703	0.796–3.643	0.170	1.405	0.479–4.122	0.536
COPD	0.233	0.071–0.768	0.017	0.458	0.099–2.117	0.318
Cardiovascular diseases	0.433	0.177–1.058	0.066	0.772	0.233–2.561	0.672
CT severity score	1.144	1.050–1.245	0.002	1.117	0.920–1.357	0.263
GGO volume (cm^3^)	1.003	1.001–1.006	0.018	0.996	0.984–1.007	0.440
GGO volume percentage (%)	1.113	1.040–1.191	0.002	0.983	0.741–1.305	0.907
Consolidation volume (cm^3^)	1.044	1.023–1.065	< 0.001	1.053	1.015–1.091	0.006
Consolidation volume percentage (%)	1.398	1.120–1.745	0.003	0.955	0.684–1.332	0.785
Clinical outcome#						
Age	1.068	1.029–1.108	0.001	1.054	1.011–1.099	0.014
Diabetes	0.290	0.120–0.698	0.006	0.313	0.111–0.886	0.029
Hypertension	1.552	0.700–3.438	0.279	–	–	–
COPD	0.495	0.146–1.683	0.260	–	–	–
Cardiovascular diseases	2.467	0.991–6.141	0.052	0.686	0.227–2.076	0.505
CT severity score	1.096	1.009–1.191	0.031	1.094	0.917–1.304	0.319
GGO volume (cm^3^)	1.002	0.999–1.004	0.189	1.001	0.992–1.010	0.853
GGO volume percentage (%)	1.064	0.998–1.134	0.058	0.896	0.715–1.122	0.338
Consolidation volume (cm^3^)	1.020	1.006–1.035	0.007	1.020	0.994–1.047	0.138
Consolidation volume percentage (%)	1.160	0.987–1.362	0.071	1.015	0.785–1.314	0.907

*AUC* area under curve, *CT* computed tomography, *CI* confidence interval, *COPD* Chronic obstructive lung disease, *GGO* ground glass opacity, *ROC* receiver operating characteristic.

*Disease severity progression was defined as the prediction of progress-to-severe group.

^#^Clinical outcome was defined as the prediction of occurrence of critical events (a. respiratory failure and need mechanical assistance; b. shock; c. “extra pulmonary” organ failure, d. ICU stay).

In terms of prognosis evaluation, 34 patients experienced the occurrence of at least one critical event. Similar to the above results, consolidation volume and consolidation volume percentage had the largest AUC in discriminating patients with and without critical events (Table 3). However, according to multivariate analysis, only age and diabetes were the significant predictors for unfavorable clinical outcomes (Table 4).

## Discussion

The main finding of the current study confirmed the value of AI assisted lesion quantification for prediction of disease severity progression. The consolidation volume on initial chest CT was the strongest predictor among all CT-derived parameters and larger consolidation volume was associated with unfavorable clinical outcome.

Chest CT is considered one pivotal diagnostic approach in the management of COVID-19 infection for detection of pulmonary involvement and serial follow-up of disease course^16^. In addition to visual diagnosis, latest technical development in the field of AI enables automatic lesion quantification using preset attenuation thresholds^17^. According to the current findings, this AI based quantification was not only beneficial for disease diagnosis but also for prognosis evaluation. Compared to conventional clinical characteristics, consolidation volume on initial chest CT was the strongest predictor for progress-to-severe group. Although GGO is the commonest finding on chest CT, consolidation has been reported to be more frequently presented in cases of severe and critical type^18,19^. Similar to other types of viral pneumonia, the underlying pathology of consolidation in the setting of COVID-19 could correlated to the complete filling of alveoli by inflammatory exudation^20^. When necrotizing bronchitis and diffuse alveolar damage occurs in the setting of viral pneumonia, the ventilation function is seriously compromised^21^. Therefore, it is conceivable that patients with larger area of consolidation on initial chest CT are more likely to progress to severe or critical clinical conditions.

Another important finding of the present study was the superiority of AI assisted quantification over conventional CT severity score for prediction of disease progression. The semi-quantitative CT score was first introduced in the evaluation of disease severity of severe acute respiratory syndrome^12^. However, this score does not take different lesion components, such as consolidation and GGO, into account. According to the current results, consolidation volume carried more significant predictive value that did GGO volume. Further, CT severity score roughly assesses the overall extent of disease according to involved zones. It is unable to distinguish mildly discrepant lesion severity when involvement difference is less than 25% within one zone. The latter one can only be evaluated by AI-assisted quantification. Thus, precise evaluation of absolute quantification based on different lesion characteristics is more helpful than CT semi-quantitative score in terms of risk stratification in patients with COVID-19. Similar findings can also be observed from one previous AI-based chest CT study, which found that percentage of consolidation volume and other CT quantified lesion parameters can early and non-invasively predict the progression to severe illness^22^.

In terms of the clinical outcome evaluation, consolidation volume had the highest diagnostic accuracy among all imaging parameters to identify patients who eventually experienced critical clinical events. This was in line with one previous semi-quantitative chest CT study that the severe extent of parenchymal involvement was associated with poor clinical outcome^23^. However, according to multivariate analysis, only age and diabetes remained as the significant predictor for poor prognosis in our study. This could be potentially ascribed to the limited number of total included patients. Future studies with larger sample size may help to determine the predictive value of consolidation volume for critical clinical events.

Despite of the above promising findings, the present study has several limitations. First, the current study did not employ radiomics analysis, which allows extraction of a large number of quantitative features from medical images for diagnosis and prognosis evaluation^24,25^. In addition, the sample size of the present study was not large enough to include more clinical characteristics and imaging features into multivariate analysis. Future studies with more included patients are warranted to validate the current results. Finally, the AI algorithm used in the current study was not able to identify other radiological findings, such as “crazy paving” and “bronchial wall thickening” in the analysis. It would be helpful to include those findings in future upgraded AI approach to further improve the performance of risk stratification.

In conclusion, consolidation volume quantified on initial chest CT was the strongest predictor for disease severity progression and larger consolidation volume was associated with unfavorable clinical outcome. AI-assisted lesion quantification was helpful for risk stratification and prognosis evaluation in patients with COVID-19.

## Supplementary Information

Supplementary Information

## Data Availability

Anonymous chest CT data will be available for review only upon reasonable request from the corresponding author. However, the data cannot be made public to maintain patients’ privacy and local legal reasons.

## Abbreviations

AI: Artificial intelligence
COVID-19: Coronavirus disease 2019
CT: Computed tomography
GGO: Ground glass opacity
RT-PCR: Reverse transcription polymerase chain reaction
