# Supplementary material for: Prediction of disease progression in patients with COVID-19 by artificial intelligence assisted lesion quantification
Source: Sci Rep. 2020 Dec 16;10:22083. doi: 10.1038/s41598-020-79097-1 (PMC7745019; doi:10.1038/s41598-020-79097-1)
Supplement: Supplementary file 1 — Supplementary Information [file 41598_2020_79097_MOESM1_ESM.docx]

Prediction of disease progression in patients with COVID-19 by artificial intelligence assisted lesion quantification

Yuehua Li^1^ M.D., Kai Shang^1^ M.D., Wei Bian^2^ M.D., Li He^3^ M.D., Ying Fan^2^ M.D., Tao Ren^3^ M.D., Jiayin Zhang^1*^ M.D.,

^1^ Institute of Diagnostic and Interventional Radiology, Shanghai Jiao Tong University Affiliated Sixth People’s Hospital, #600, Yishan Rd, Shanghai, China

^2^ Department of Respiratory Medicine, Shanghai Jiao Tong University Affiliated Sixth People’s Hospital, #600, Yishan Rd, Shanghai, China

^3^ Department of Nephrology, Shanghai Jiao Tong University Affiliated Sixth People’s Hospital, #600, Yishan Rd, Shanghai, China

Details of deep convolutional neural network based on residual structure UNet

UNet is one of the most classic architectures in deep learning. It includes a series of downsample steps and also a series of upsample steps [1]. For convolution kernels are sensitive to features in a specific range, downsample steps actually scale down features so the whole pipeline can detect features from low level to high level. Specifically, in our research, the texture of pneumonia, shape, density and other characteristics can vary. Multi-scale feature extraction by downsample steps in Unet can improve the pipeline performance.

After feature extraction, upsample steps merge features from different scales by scaling up features in smaller shapes and then give a final result. Notably, when merging features, UNet architecture employs residual connections from the downsample side to the upsample side. These residual connections prevent gradient vanishing, so the whole network is easier to train. Moreover, the residual connections ensure the information will not be lost during downsample steps.

Reference:

1. Rong Y, et al. Deriving external forces via convolutional neural networks for biomedical image segmentation. *Biomed Opt Express*.**10**:3800-3814 (2019).
